# Trauma-informed palliative care is needed: A call for implementation and research

**DOI:** 10.1177/02692163231206998

**Published:** 2023-10-30

**Authors:** Janet M de Groot, Dwain C Fehon, Lynn Calman, Danielle S Miller, Andrea Feldstain

**Affiliations:** 1 Department of Psychiatry, Cumming School of Medicine, University of Calgary, Calgary, AB, Canada; 2 Department of Psychosocial and Rehabilitation Oncology, Tom Baker Cancer Centre, Calgary, AB, Canada; 3 Department of Psychiatry, Yale School of Medicine, New Haven, CT, USA; 4 Smilow Cancer Hospital, New Haven, CT, USA; 5 Centre for Psychosocial Research in Cancer, School of Health Sciences, University of Southampton, Southampton, UK; 6 Department of Counseling Psychology, Ball State University, Muncie, IN, USA; 7 Department of Oncology, Cumming School of Medicine, University of Calgary, Calgary, AB, Canada

Trauma-informed palliative care is an emerging field^1,2^ that may enhance the care of people living with life-threatening and life-limiting illnesses, with pre-existing trauma and/or recent trauma experiences, that also extends to their primary caregivers and healthcare providers. Trauma in this model includes adverse childhood events, discrimination and racism and severe-life threatening events. Trauma-informed care, defined by the Substance Abuse and Mental Health Services Administration, includes four assumptions and six principles.^3^ The foundational assumptions recognize the pervasiveness of trauma and its adverse impact on physical and mental health.^3^ Additionally, this understanding of trauma is integrated into policy, practice and procedures, with attention to avoiding re-traumatization.^3^ The six principles of trauma-informed care encompass safety, trustworthiness and transparency, peer support, collaboration and mutuality, empowerment and attention to historical, cultural and gendered contexts.^3^ While Brown et al.^2^ have outlined how clinical practice guidelines for palliative care align with trauma-informed care a research agenda is needed to identify priorities and help define best practices for trauma-informed palliative care.^4^

Traumatic experiences may influence an individuals’ perceptions, and limit integration of emotional and cognitive information.^5^ Subsequently, new information or stressful experiences may be identified as threatening and contribute to distressing behavioural responses,^5^ including avoidance, dissociation and aggression. Unlike psychological treatments for trauma, trauma-informed care does not directly address the source of trauma and recognizes that trauma resolution occurs through multiple pathways.^4^ Healthcare providers, aware of trauma presentations, focus on building trust and effective communication. Perceived social support and social inclusion are pivotal in moderating the development and maintenance of post-traumatic stress disorder symptoms.^6^ In settings of psychological safety, strong social attachments with family, friends and healthcare providers may facilitate processing trauma-related emotions and cognitions promoting connectedness for resilience.^6^ A focus on collaboration, empowerment and consideration of cultural factors may involve seeking direction from patients about preferences for bodily care, communication and consistency of care providers. Greater understanding of the needs of minoritized communities, (including people who identify as Indigenous, or with racial, ethnic and sexual or gendered minorities or with intersectional identities) that require palliative care is necessary, due to historical trauma, discrimination and structural inequities present within our healthcare systems. An inclusive person-centred approach may enhance trust in palliative care providers, minimizing re-traumatization.

A trauma-informed palliative care rationale includes theoretical, epidemiological and treatment considerations. Palliative medicine’s goal is reducing physical, emotional and spiritual suffering while maintaining quality of life for individuals with life-threatening and life-limiting illnesses. From a psychological perspective, the very real existential threats imposed by a life-limiting illness may trigger memories of prior traumatic events, thus exacerbating acute traumatic stress symptoms.^7^ Indeed, up to 80% of people with advanced, recurrent cancer experience post-traumatic stress symptoms compared to 20% of patients with early-stage cancer.^8^ People with life-limiting illnesses may also experience healthcare-induced trauma resulting from painful surgeries, procedures, medication adverse effects, ICU admissions and ER visits. These new stressors, combined with declining physical function may induce death anxiety or reactivate memories of prior trauma, influencing patients’ acceptance, interact with or refusal of palliative care.^1^ Additionally individuals with post-traumatic stress disorder approaching end of life, have a higher symptom burden and an increased likelihood of treatment with anti-psychotic medications.^7^ Specific populations that may particularly benefit from trauma-informed palliative care include but are not limited to: individuals with a history of childhood abuse or other adverse childhood experiences, war veterans with post-traumatic stress disorder, persons displaced by conflict with its associated chaos, children and youth with life-threatening illnesses and their primary caregivers,^9^ and individuals admitted to the ICU with life-threatening reactions to cancer treatment.

## Healthcare provider, organizational and institutional implications

Effective trauma-informed care requires integration throughout an institution, encompassing leadership and healthcare providers. Well-defined trauma-informed care includes describing actionable steps and providing examples for each principle.^4^ Trauma-informed interprofessional teams can enhance collaboration to address patients’ psychological and medical needs.^4^ Healthcare providers, especially frontline nursing staff, require education to recognize traumatic behavioural presentations to respond helpfully, considering the psychological trauma and medical aspects of palliative care.^10^ Outpatient settings are conducive to establishing therapeutic relationships that facilitate patient’s and family’s emotional and perceptual processing related to life-limiting illnesses.^11^ Consultation with psychosocial palliative care clinicians may enhance understanding and support for complex patient presentations.^11^

It is essential to be mindful that healthcare providers themselves may be affected by pre-existing trauma or experience vicarious trauma in providing palliative care.^6^ Organizational support for trauma-informed care practices can enhance staff effectiveness and morale.^6^

## Trauma informed care and narrative medicine

Narrative medicine, which explores illness experiences through storytelling complements trauma-informed care. It facilitates the empowerment principle, as patients share their thoughts and feelings in a manner that biomedical approaches may overlook, potentially fostering collaboration between patients and providers.^12^ During the COVID-19 pandemic, the RISE (Resilience, Inspiration, Storytelling and Empathy) online program utilized storytelling as a trauma-informed response for meaning-making benefiting global health providers.^13^ Patient and healthcare provider narratives may enrich healthcare education.

## Future directions and research

Trauma-informed care is still a relatively new concept within the palliative care field. Validated, evidence-based approaches for staff training, trauma assessment, program development and intervention in palliative care are needed. To accomplish this, we propose that funding resources and a research agenda be developed that encompasses education, program development, program implementation and organizational policy.

### Education

Education is paramount for palliative healthcare providers to heighten their awareness of how patients present with trauma and to adapt care provision to ensure psychological safety and trust.^14^ Training in cultural humility, understanding trauma and its symptoms, knowledge of trauma-informed care and education on the interpersonal qualities of compassion, patience, respect, sensitivity and non-judgement are needed to facilitate psychological safety. Educational tools such as videos featuring standardized patients with varying trauma presentations can be valuable for identifying trauma-related presentations, addressing trauma disclosures and teaching grounding techniques.^10^ Staff education may include train the trainer sessions, staff workshops and champions to act as peer mentors.^14^ To further enhance knowledge, skills and attitudes of practicing clinicians, communities of practice may deepen or create new knowledge through case discussions and support collective moral resilience.^15^ Going forward, trauma-informed care education may be essential for undergraduate and postgraduate trainees across mental health disciplines, medicine and nursing.

### Assessment

A universal precaution or standard screening approach to assess trauma in primary care and pediatric settings has been recommended.^16^ However, validated assessment tools to screen for trauma, especially in the elderly and for those in palliative/hospice settings, are lacking. Research is needed to develop appropriate tools and screening techniques that can be effectively integrated into palliative care practice. Since some patients with palliative and hospice settings may lack the ability to provide their own self-report, screening techniques should include the assessment of observed behaviour and validated tools to be completed by caregiver/family on behalf of the patient are also needed.

### Intervention

While evidence-based treatments for trauma and PTSD (e.g., CBT and EMDR) exist within the field of mental health, it is imperative to develop brief evidence-based interventions and approaches to alleviate trauma symptoms in hospice and palliative care settings. Novel interdisciplinary interventions could be created by palliative care researchers linked with experts in trauma-informed interventions and importantly, collaborating with people who have lived experience of post-traumatic stress disorder or acute stress disorders. Trauma-informed interventions in the context of palliative care may include breathwork, grounding techniques, body awareness, relaxation, visualization, mindfulness meditation and expressive therapies (e.g., art, music and journaling). Staff training should emphasize skills to avoid re-traumatizing patients. Additionally, interventions to reduce traumatization and to maintain the well-being of family caregivers and healthcare providers are needed.

### Program development, implementation and evaluation

Research is necessary to examine factors that facilitate a trauma-informed palliative care approach. We require research designs that can explore the complexity of the intersection between palliative care and trauma-informed care. Qualitative research is needed to understand the personal impact and meaning of trauma in the palliative care setting, including its effects on patients, caregivers and professionals. These rich insights can support identification of meaningful outcomes for trauma-informed care. Program development studies should include identifying an implementation framework, relevant program settings and co-design initiatives with leaders, staff, patient and family stakeholders to facilitate a trauma-informed approach.^14^

Implementation science research includes evaluation of implementation strategies, and assessment of clinical, staff and economic outcomes.^14^ The implementation of a trauma-informed approach should include assessing staff perceptions of the relevance of a trauma-informed approach to healthcare, support for staff and evidence-based practice.^14^ Evaluation of programmes will require innovative approaches that acknowledge the context and complexity of the area. Outcome assessment may include patient quality of life, patient experience, medication use to manage trauma-related symptoms and length of stay in inpatient palliative care units. Staff outcomes may include staff skills and confidence, as well as staff’s own perceptions of psychological safety and trauma. Barriers and facilitators to implementation are important.

### Organizational policy

The development of policies, procedures and protocols is essential to achieving clinical excellence. Integrating trauma-informed palliative care into policy and practice necessitates organizational commitment, program development, staff education and implementation strategies as well as dedicated funding. We propose that a commitment to trauma-informed care, which resists re-traumatization, be included within the mission and values statements of all palliative care organizations. Further, trauma-informed care includes all aspects of palliative care (e.g. patient/family, provider, clinical teams organizational leadership and healthcare financing structures). Studies which examine the barriers and enablers for the inclusion of organizational policy are needed.

For sustainability and institutional buy-in, research is needed to study the cost-effectiveness and cost-benefits of implementing a trauma-informed approach. Along these lines, policies and procedures that attend to support needs of healthcare workers, help prevent burn-out and aid in staff retention are especially needed.

## Conclusion

Given our understanding that trauma is ubiquitous and often adversely impacts an individual’s physical and mental health particularly with advanced illness, a trauma-informed approach to palliative care is clearly needed. Taking into account the impact of both pre-existing and medically-induced psychological trauma benefits patients, primary caregivers and healthcare staff. The inclusion of a trauma-informed care approach into palliative care is entirely consistent with the values and goals of palliative care (patient-centered care, respect for patient preferences/priorities, reducing suffering and improving quality of life). Given the growing efforts to develop trauma-informed care approaches in healthcare in general, and in palliative care in particular, funding resources and a research agenda are urgently needed to help move the field forward in effective and sustainable ways that serve not only patients and their families, but also serves the needs of healthcare and palliative care clinicians as well.
